# Earlobe Reconstruction With an Anteriorly Based Postauricular Flap

**Published:** 2020-04-17

**Authors:** Jeremy Chang, Caroline Awh, Charles A. Riccio, Petros Konofaos

**Affiliations:** Department of Plastic Surgery, University of Tennessee Health Science Center, Memphis

**Keywords:** earlobe reconstruction, skin flap, earlobe avulsion, Post-auricular flap, ear reconstruction

## DESCRIPTION

A 28-year-old man presented with amputation of a 3-cm portion of his right earlobe secondary to an altercation with his girlfriend. The earlobe was reattached in the emergency department, but follow-up at 1 week revealed necrotic tissue. The patient subsequently chose to undergo local flap reconstruction. An approximately 8-mm flap was incised, based on anterior blood supply, and inset was carried out in a circumferential fashion from the distal corner.

## QUESTIONS

1. What are indications and contraindications for earlobe reconstruction?
2. What are the different types of earlobe reconstruction for full-thickness lacerations?
3. How was this particular flap raised?
4. What are the anatomical structures to be aware of in earlobe reconstruction and potential complications of the procedure?

## DISCUSSION

The preferred treatment of full-thickness earlobe lacerations is primary closure within 24 hours of the initial insult. Timing is imperative for treatment. Cartilage forming the ear is avascular and requires appropriate fixation with perichondrium for survival.^1^ Exposed cartilage and delayed presentation drastically increase risk of infection, chondritis, and necrosis.^2^ Signs of inflammation represent a definitive contraindication for closure and must be dealt with before attempting reconstruction.

Various techniques have been reported for total earlobe repair, using 1- or 2-stage procedures to create a skin flap from adjacent tissue. Skin flaps are primarily taken from the preauriclar, infra-auricular, retroauricular, or retromandibular areas or from the auricular surface. The earlobe is supplied by vessels that arise from the posterior aspect of the posterior auricular artery and the anterior auricular branches of the superficial temporal artery.^3^ Choice of donor site for the skin flap is dependent on the viability of the adjacent tissues and perforators, residual deformity, and aesthetics of postoperative scarring. The earlobe can be reconstructed using a doubled-over single or bilobed flap.^4^ This flap consists of 2 wings below the auricular defect, supplied by an inferior base, located on both the pre- and postauricular folds, which are brought together to rebuild the new earlobe.^5^ A disadvantage to the bilobed flap is that it is only able to reconstruct smaller defects. Some techniques are 2-stage procedures that incorporate a cutaneous or cartilaginous graft to the flap based on the extent of the defect.^6^ One such 2-step method involves insertion of a cartilaginous graft into a dissected pocket and mobilization of the auricle with full-thickness skin grafting.^6^

The flap for the abovementioned case was raised from the posterior auricular area. Choice of donor tissue from the posterior auricular area was dictated by viability of tissue after initial debridement. Debridement of necrotic tissue was followed by mobilization of an 8-mm retroauricular donor flap utilizing vascular pedicles from perforators of the anterior auricular artery. Insetting of the flap to the lesion was completed beginning from the distal corner of the lesion using 5-0 Vicryl Rapide and finished in a circumferential fashion about the ear. Cartilage was preferentially closed with a braided absorbable suture while skin was closed with 6-0 monofilament.^1^

Potential complications of earlobe reconstruction include hematoma formation, “cauliflower” ear deformity, and damage to vital neurovascular structures. Hematomas may form in the potential space between cartilage and perichondrium.^1^ “Cauliflower” ear develops due to lack of perichondrium adherence to cartilage leading to abnormal cartilage production and fibrotic calcified deformity. The sensory nerves that supply the earlobe are the posterior branch of the great auricular nerve (branch from cervical plexus) and the auriculotemporal nerve (branch of V3). These nerves are at risk for injury during earlobe reconstruction; however, studies suggest that although patients experience abnormal sensation after great auricular nerve sacrifice, it decreases significantly over time to ultimately provide little discomfort to the patient.^7^

Overall, the ear represents a challenging proposition for obtaining a cosmetically appealing result while preventing the need for additional intervention. The cartilaginous nature and delicacy of vital structures further lend to the difficulty of reconstruction in this region. However, literature highlights approaches to achieve a functional and aesthetic outcome.

## Figures and Tables

**Figure 1:**
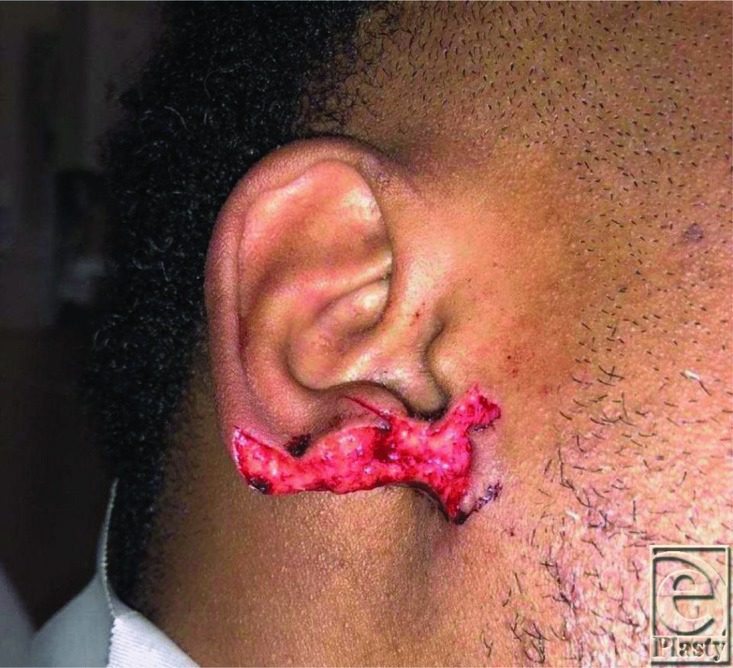
Preoperative: Right earlobe amputation prior to reconstruction.

**Figure 2:**
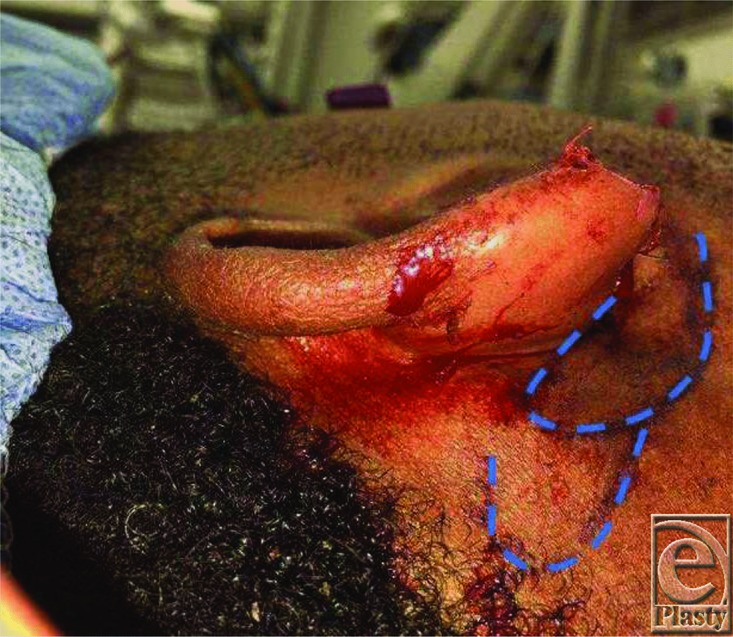
Perioperative: Markings for the local anteriorly based postauricular flap.

**Figure 3:**
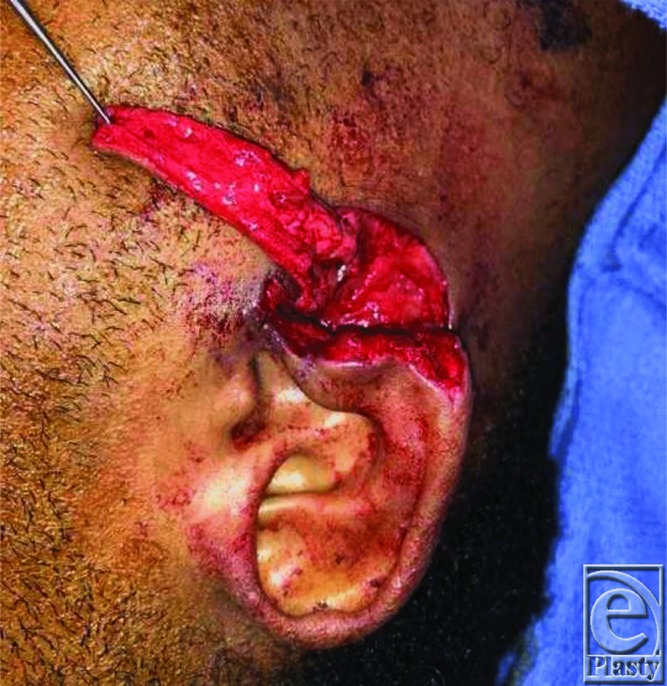
Perioperative: Mobilization of flap.

**Figure 4:**
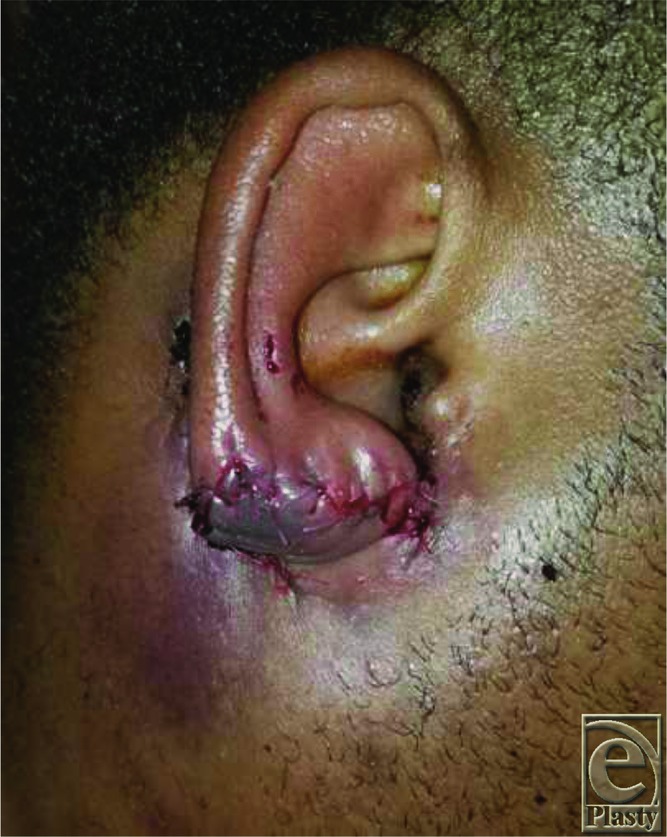
Postoperative: Distal corner of the flap was inset at the distal aspect of the defect and inset was carried out in a circumferential fashion.

**Figure 5:**
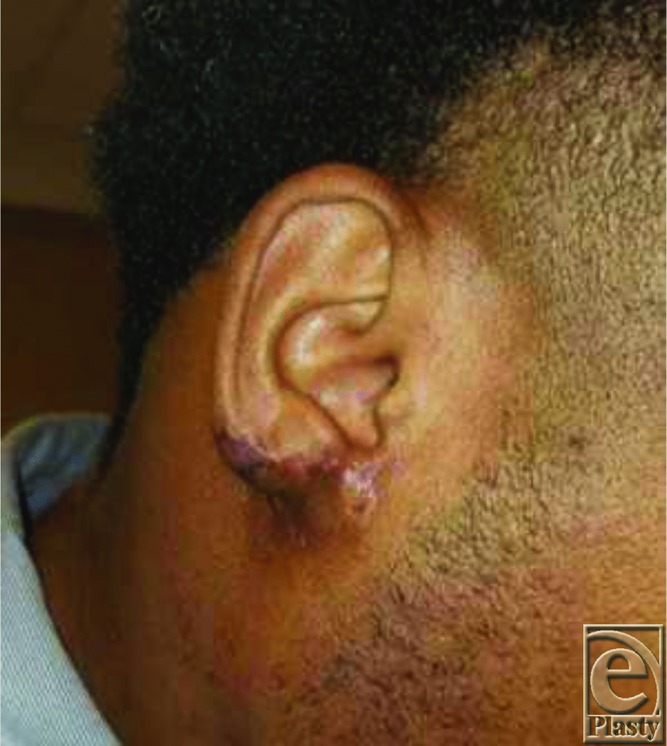
Six weeks postoperative.
